# Computer-Aided Design/Computer-Aided Manufacturing of Customized Abutment for Rehabilitating a Malpositioned Implant Using Digital Flow: A Case Report

**DOI:** 10.3390/healthcare11182472

**Published:** 2023-09-06

**Authors:** José Henrique Lopo Barros, Murilo Navarro de Oliveira, Igor Oliveiros Cardoso, Guilherme José Pimentel Lopes de Oliveira, Célio Jesus do Prado, Flávio Domingues Das Neves

**Affiliations:** 1 Department of Occlusion, Fixed Prosthodontics and Dental Materials, School of Dentistry, Federal University of Uberlandia, Uberlandia 38405-320, MG, Brazil; celio.prado@ufu.br; 2 School of Dentistry, University Center—UNIFAE, São João da Boa Vista 13870-377, SP, Brazil; murilo_n_o@hotmail.com; 3 College of Dentistry, University Center of Triangle (UNITRI), Uberlandia 38411-849, MG, Brazil; igorcardoso@hotmail.com; 4 Department of Periodontology and Implantology, School of Dentistry, Federal University of Uberlandia, Uberlandia 38405-320, MG, Brazil; guilherme.lopesoliveira@ufu.br

**Keywords:** CAD/CAM technology, digital flow, tooth fracture, dental implant, customized abutment (CAD/CAM)

## Abstract

This study presented a rehabilitation option for malpositioned implants; this involved obtaining their position and inclination through intraoral scanning, and producing a customized abutment with CAD/CAM technology. The patient in this case report presented a root fracture in tooth 21 and was subjected to extraction, implant installation, and immediate provisional prosthesis. The implant was installed with a distal inclination due to anatomical limitations. After osseointegration, an intraoral scanning transfer provided a digital model (file extension .stl), which reproduced the implant’s position and inclination. Then, the file was sent so that a customized abutment (CAD/CAM) could be manufactured, promoting the final rehabilitation of the case; this allowed for good hygiene, load distribution in the dynamic interocclusal relationship, and favorable esthetics, whereas many would otherwise recommend implant removal. The result presented lower costs, a shorter time frame, and a lower morbidity for the patient.

## 1. Introduction

Malpositioning is among the more substantial problems in dental implant rehabilitation [1]. Implants installed in inadequate positions may reduce the longevity of prosthetic restorations [2,3]. Proper planning can help to install dental implants in the optimal three-dimensional positions, allowing the production of a correct prosthesis on an implant in order to obtain satisfactory functional and esthetic results [4]. It is worth noting that any dental prosthesis must restore its function according to dental-arch positioning, which mainly includes masticatory and esthetic aspects. It must also be long-lived, which is achieved through good hygiene and masticatory effort distribution. Customized angled abutments may represent an alternative when installed implants are incorrectly angled, more inclined than prefabricated angled abutments [5,6]. However, despite solving the technical problem, analyzing the biomechanics of the implant insertion area is prudent because compromising good hygiene and/or masticatory effort distribution may harm the longevity of the procedure. Studies have constantly focused on correct implant positioning, culminating in guided surgeries, and, more recently, navigated and robotic surgeries [7,8]. Such measures have highly optimized the satisfactory definition of the position and inclination of dental implants. However, borderline situations with extensive bone losses associated with the presence of major anatomical structures may further complicate implant position and inclination. This issue does not greatly impact multiple splinted cases, and effort distribution is even favorable for some inclinations [9]. Thus, single teeth become concerning due to masticatory effort distribution and esthetic areas regarding the preservation and reproduction of the adjacent mucogingival architecture [10]. Nonetheless, recent studies have compared guided and free-hand surgeries, showing that many professionals still choose the latter technique [11,12,13].

The development and use of CAI/CAD/CAM (Computer-Aided Imagining/Computer-Aided Design/Computer-Aided Manufacturing) in dentistry has provided opportunities for dentists to present fast and effective solutions for different prosthetic rehabilitation scenarios, including implant dentistry [14]. This technology allows for the operator to create prosthetic restorations, and, with an intraoral scan, obtain an image reproducing the three-dimensional position of the implant or abutment in software. This allows for the creation of a customized abutment according to specific patient needs [15,16], fixing incorrectly installed implants without esthetic damage for the final rehabilitation [17]. Each case must be analyzed under esthetic, masticatory effort distribution, and hygiene criteria. Although abutments have been manufactured with the CAD/CAM system for over 20 years [18], the development and commercial availability of several intraoral scan cameras (from transfers for intraoral scanning) and software that generate virtual models have revolutionized this practice. Digital flow facilitates clinical practice, reducing steps and simplifying laboratory procedures [14]. The process becomes more convenient for dentists and patients due to the possibility of performing multiple scans, analyzing the image in real-time, and preventing potential unwanted situations for patients with conventional impressions, such as the risk of suffocation and gagging, nausea, and unpleasant tastes [15,16]. Therefore, this study presented a prosthetic solution using digital flow on an implant installed in the anterior maxillary region in an angled position without removing and installing a new implant. The duly informed patient chose this solution, considering it adequate and less risky than the proposal of grafting and a more satisfactory implant installation.

## 2. Case Report

**Figure 1:**
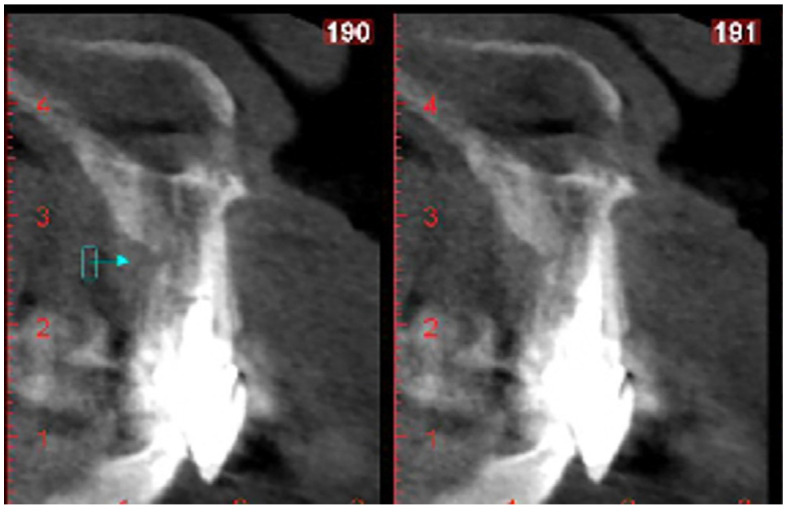
Tomography image of the fractured tooth.

A 50-year-old male patient attended the dental clinic for the prosthetic rehabilitation of tooth 21 with a fracture diagnosis. Clinical and radiographic analyses showed a root fracture in element 21 caused by a short metallic intra-radicular retainer. The patient had a history of fractures in the upper anterior teeth for the same reported reason. A tomography of the area was requested, presenting a distalized root and a displaced incisive foramen relative to the midline, occupying the mesial crown region of the referred tooth, thus hindering an immediate implant installation in an ideal position (Figure 1).

Hence, two treatment possibilities were presented and discussed with the patient. One of the options for optimal implant placement would require extracting tooth 21, waiting for bone repair, and analyzing the need for a buccal graft. The patient would wear a removable or direct adhesive prosthesis during this time. It is worth mentioning that the presence of ceramic crowns on neighboring teeth might complicate the installation of a satisfactory adhesive prosthesis. The other option was extraction with immediate implantation; this had a good chance of immediate loading but with a distalized implant relative to an ideal implant, which would be corrected in the prosthetic abutment without impairing function. The duly informed patient preferred immediate installation, and the implant was installed distally to the three-dimensional center of the clinical crown space for rehabilitation. Therefore, an extraction of tooth 21 was planned with free-hand surgery for implant installation and possibly immediate load. The tomographic analysis showed that the neighboring tooth was a reference for buccolingual inclination and the long clinical crown axes of elements 11 and 21 of the mesiodistal parallelism. The achieved primary stability was compatible with immediate loading—45 Ncm [19]. However, at the end of implant installation, a higher-than-expected distal inclination occurred, raising the hypothesis of implant removal or even a repositioning attempt, but with a high likelihood of losing stability and an immediate-load opportunity. The decision to maintain it may not be unanimous but must be made in seconds; thus, the option of guided surgery is favored in these situations. Due to professional and psychological reasons, the patient valued a stable and safe provisional prosthesis.

**Figure 2:**
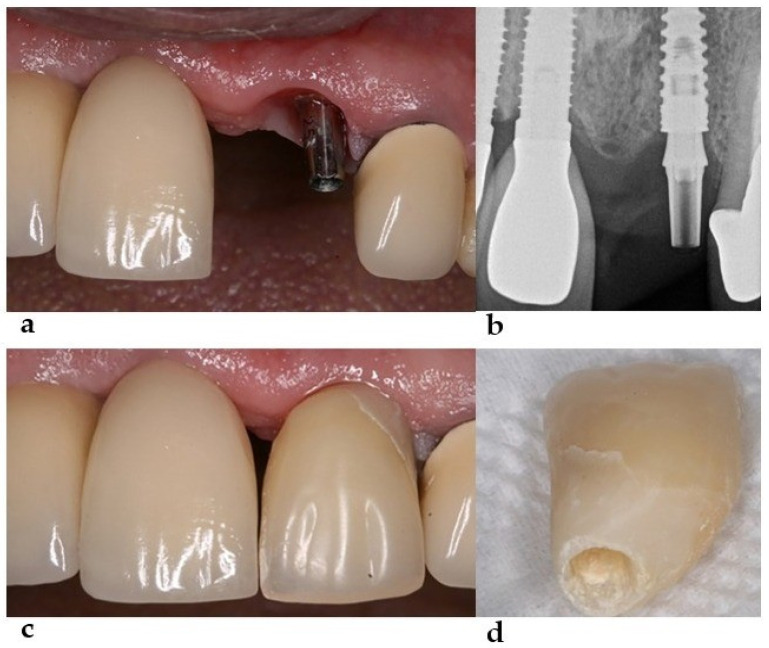
(**a**) Universal abutment installed over the implant, displaying the implant inclination. (**b**) Radiographic aspect of the installed abutment. (**c**) Provisional restoration over the abutment. (**d**) Provisional restoration showing a low thickness at the distal region.

The torque was compatible with an immediate provisional installation, which led to the decision to maintain the implant in place, allowing for such an installation under immediate loading. After installing the dental implant (3.75 × 11, Drive CM, Neodent, Curitiba, Brazil), the abutment for the cemented (Universal Abutment—3.3 × 6 × 2.5 mm—Neodent) and provisional prosthesis was installed (Figure 2). The provisional prosthesis was removed after osseointegration. The thickness of its distal wall was thin, and the space between the abutment and adjacent tooth was insufficient for the correct volume of ceramics in the region, evidencing the need for an angled abutment (Figure 2). Unlike the initial plan, commercially available prefabricated angled abutments would not solve the problem caused by excessive distal inclination. Customized abutments can provide a crown with adequate thickness and preserve the mucogingival architecture, resulting in more favorable esthetics.

**Figure 3:**
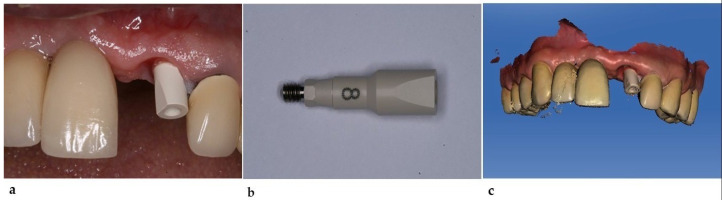
(**a**) Scan body positioned on the mouth. (**b**) Scan body. (**c**) Digital image obtained after the intra-oral scanning.

Implant position and inclination were obtained through an intraoral scan (Omnicam, Cerec Sirona, Charlotte, NC, USA); we used a Morse taper implant scanbody (Neodent^®^ 108.211/Transfer for intraoral scanning—MT implant) which was screwed directly into the implant, providing a digital model with the 3D positioning of the implant, peri-implant tissues, and adjacent teeth (Figure 3).

**Figure 4:**
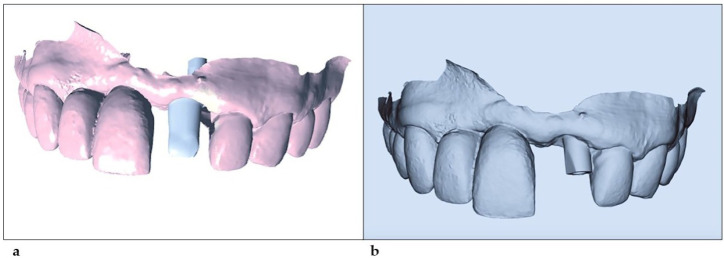
(**a**) Image received by the company for the approval and manufacture of the abutment. (**b**) .stl file sent to Neodent.

After obtaining the files of the lower arch (antagonist, intermaxillary registration) and upper arch with the intraoral scanning transfer (working pre-model in Cerec’s CAD 4.6 software), they were exported in .stl format and sent to the laboratory (D-Lab Curitiba-Brasil) for planning and manufacturing the customized titanium abutment (Figure 4).

**Figure 5:**
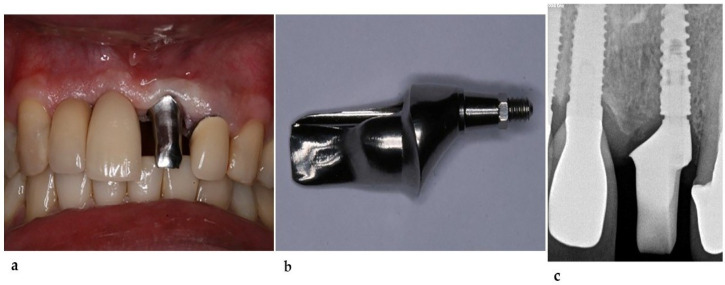
(**a**) Customized abutment positioned in mouth. (**b**) Customized abutment. (**c**) Radiographic aspect of the abutment over the implant, showing the component adaptation.

This arrangement was sent by e-mail for verification after approval, mastered on a milling machine (DMG Ultrasonic 10, Idar-Oberstein, Germany), and was ready for mouth-testing in three days. The process in question was possible because the used implant system provides the titanium block (AG support—Neodent^®^); additionally, the implant-fitting joint was already machined and the abutment design was customized. The abutment was then tried on and installed (Figure 5). The implant’s position and inclination corrections were clinically confirmed, favoring prosthetic spaces, mainly distally.

**Figure 6:**
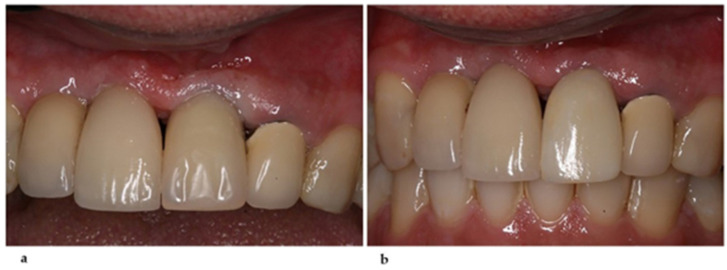
(**a**) Provisional restoration over the customized abutment. (**b**) Final restoration after five years of follow-up.

**Figure 7:**
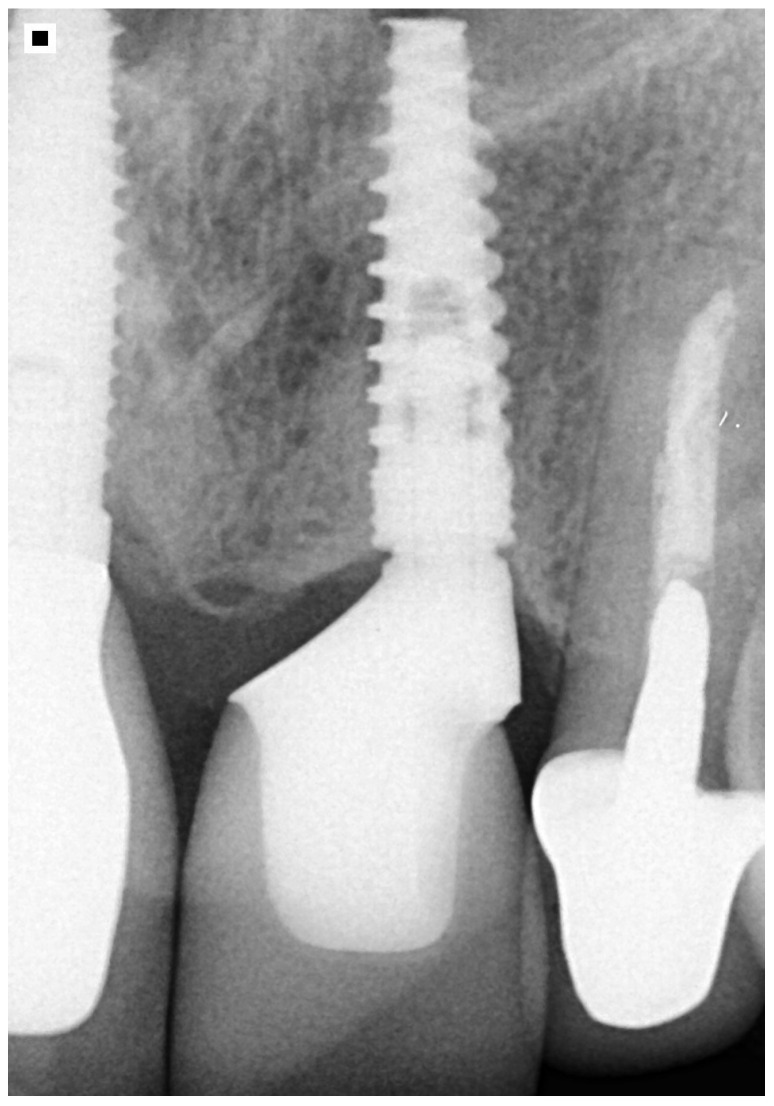
Five-year follow-up.

A radiographic examination was performed with the installed abutment to verify its adaptation, and a new provisional prosthesis was made using a stock tooth in acrylic resin. Considering the favorable gingival biotype and abutment cervical end similar to crowns on teeth, after a week of gingival conditioning with the provisional restoration, the molding step started [20]. A 000 retractor wire (UltraPak, Ultradent, INC Products, South Jordan, UT, USA) was initially inserted in the gingival sulcus, and a second 0 retractor wire (UltraPak, Ultradent, Products INC, USA) was introduced into this sulcus for horizontal retraction. The Omnican scanner (Cerec Sirona) performed the digital impression of the dental preparation, antagonist, and interocclusal registration. The virtual restoration was made in the software (Cerec 4.6—Sirona), and the file was sent for materialization in the CAM-milling machine (MCXL—Sirona) from a ceramic block (Empress Multi Color A2) (Figure 6). The work has since been monitored and it fulfilled its five-year requirements without harming the esthetics and/or the remaining bone (Figure 7).

## 3. Discussion

Some factors may influence the ideal installation of dental implants when planning rehabilitation, such as reabsorbed alveolar ridges and the proximity to noble anatomical structures (maxillary sinus, nasal fossa, incisive foramen, and lower alveolar nerve) [21,22]. The need to change the ideal location may compromise the parallelism, inclination, and final esthetics of implants [23]. When correct implant installation is impossible, the team must decide between correction through bone reconstruction or different prosthetic resources. Patients must be informed about possible techniques and their advantages and disadvantages so that they can consciously choose the most suitable according to their values. The growing esthetic demand of patients emphasizes the relevance of installing provisional restorations immediately after surgical implant installation, especially regarding anterior maxillary regions [24,25]. Immediate provisional restorations may be removable or fixed, and the latter can be made with retentions in adjacent teeth, directly on the implant or abutment, or adhesive ones [26,27]. The patient in this clinical case was reportedly aware of the unsatisfactory implant position, which remained as such to maintain the immediate load; the presence of ceramic crowns on adjacent teeth made it difficult to produce and maintain a fixed adhesive restoration, and the patient objected to using a removable one. The patient was instructed on the need for a primary torque which is compatible with immediate loading [19] during implant installation to fabricate a fixed provisional restoration under immediate loading. Hence, an adhesive prosthesis could be required. However, the patient highlighted that the other option, extraction before implant installation and with potential bone grafting, might extend this period further with the adhesive prosthesis.

Angled abutments are used for rehabilitating inclined implants [28]. However, they present limitations, such as the exposure of metallic braces in cases of shallow gingival depths and thin phenotypes, more technically difficult installations, and a higher cost compared to straight abutments [29,30]. Moreover, single cases cannot share efforts with other implants; additionally, an incorrect inclination may cause inadequate masticatory effort distribution, harming implant longevity in the medium and long term for either mechanical reasons, such as fractures of prosthetic components [31], or biological reasons, such as bone loss [32]. This clinical case could not use prefabricated angled abutments, as their macrogeometry could not fix the implant’s position and inclination. Thus, customized abutments machined by CAD/CAM technology were required. These abutments are usually more expensive than commercially available angled ones, take longer to be acquired, and are not provided to any system. Internal joint systems require blocks pre-machined to the joint to customize the abutment. However, the problem of using angled abutments remains, and only one inclination was customized. The anterior region made the presented treatment suggestion possible, as central incisors are responsible for disocclusion during the protrusion movement established in food incision and cutting; the anterior region does not receive loads in its long axis during this function.

Regardless of its central or distal position and inclination, the load will remain lower than in the posterior region and always toward the anterior aspect. Implants placed outside the center of the future crown are hard to clean due to the accentuated contour of the implanted prosthesis. Good hygiene can increase the chances of implant survival and can help maintain the health of peri-implant tissue. When it is impossible to install the implant in an optimal location, the team must analyze, with the patient, the hygienic condition of the future rehabilitation and decide on its feasibility, as buccal projections of the prosthetic crown invariably create favorable conditions for the appearance of constant mucositis [33]. This complicates the maintenance of malpositioned implants and invariably the need for using special cleaning techniques. The position in the described case was distalized, creating a higher extension to the mesial aspect in the direction of dental flossing, facilitating cleaning, and favoring peri-implant health maintenance. The radiograph on Figure 7, with a five-year follow-up without a history of mucositis or any other discomfort, suggests satisfactory biomechanics and the possibility of good hygiene maintenance. It is worth noting that marginal bone-ridge maintenance may be associated with implantology success [34,35]. Other situations must be analyzed individually. Although posterior areas and mesial or distal positioning do not hinder hygiene, they may compromise masticatory effort distribution (more than in the anterior region). Also, a lingual implant position in anterior and posterior areas increases the buccal volume of implant crowns, which complicates cleaning and may render the suggested proposal unfeasible.

Several modern resources have been incorporated into rehabilitation treatments, showing promising results [36]. The Computer-Aided-Imagining/Computer-Aided-Design/Computer-Aided-Manufacturing (CAI/CAD/CAM) system provides digital restoration and dental models, and has been increasingly developed within various dentistry fields, such as restorative dentistry [37,38,39,40]. The development of CAI/CAD/CAM technology for manufacturing restorations and models on teeth and implants has revolutionized dentistry, offering a new form of rehabilitation, and facilitating and speeding up the workflow [41,42,43,44]. Likewise, abutments for implanted prostheses have been customized with CAD/CAM since the early 2000s [18]. The difference in the present study is the Chairside digital flow, which does not require a physical model, making the process even faster and providing comfort to patients. Although the patient was informed and aware of the situation, the higher-than-expected inclination complicated the case further, requiring a customized abutment. This process was possible and somewhat easy due to an obtained file on implant position and inclination and the gingival profile, which allowed for milling a component that practically and quickly fixed the incorrect position and inclination of the installed implant. Three steps are required to obtain restorations, models, or customized abutments made with this technology. First is data acquisition by scanning the mold, model, or patient’s mouth, as demonstrated in this case report. The second step is data processing using software, which provides a virtual design of the structure, restoration, or abutment. This stage consists of designing and planning the work in computer software. The first two steps (data acquisition and processing) constitute CAI and CAD, respectively. Manufacturing is the third stage, called CAM. The data from the executed project are sent to a milling machine that performs the machining of parts in a significantly shorter clinical and laboratory time [43,45,46,47]. Studies have shown promising results when introducing 3D printers in the manufacturing process (CAM) [48,49,50]. However, the clinical and laboratory time did not significantly improve in the rehabilitation of the present clinical case. The digital workflow of this case provided a shorter clinical time with the patient in the chair, and higher convenience in the digital impressions than non-digital ones [51]. Moreover, the dentist may analyze and approve the digital planning and design of the customized abutment before manufacturing, according to the emergence profile and specific angulation of the implant’s position and inclination. Another advantage is allowing for different designs from those commercially available. However, as mentioned, the search for more safety in attaining a correct implant position and inclination must be emphasized because it guarantees easy cleaning and good masticatory effort distribution, allowing us to easily use abutments that are commercially available, less expensive, and that safely accelerate case conclusions. Such a finding favors guided surgeries [11,12,13], although immediate implants, even when guided, may display higher variation regarding installations in healed edges [52].

## 4. Conclusions

The present case report demonstrates the rehabilitation of a patient with a single implant installed in a more distalized position and inclination in a highly esthetically demanded area. The rehabilitation proposal was only possible because the analyzed tooth was in the anterior region and the position of the potential implant installation did not hinder cleaning. Other situations must be analyzed individually, and poor masticatory effort distribution and/or hygiene impossibility will render the suggested proposal unfeasible. However, because the scenario was favorable, the proposal showed a satisfactory result with a lower cost, shorter time frame, and lower morbidity for the patient.

## Data Availability

All data generated are reported in the article.

## References

[1] Yilmaz B., Ozcelik B.T., Sarantopoulos D.M., McGlumphy E. (2012). Importance of CT scans in diagnosing symptoms from misplaced implants. Implant. Dent..

[2] Askary A.S., Meffert R.M., Griffin T. (1999). Why do dental implants fail?. Part I. Implant. Dent..

[3] Askary A.S., Meffert R.M., Griffin T. (1999). Why do dental implants fail? Part II. Implant. Dent..

[4] Grunder U., Gracis S., Capelli M. (2005). Influence of 3-D bone to implant relationship on esthetic. Int. J. Periodontics Restor. Dent..

[5] Gualini F., Berglundh T. (2003). Immunohistochemical characteristics of inflammatory lesions at implants. J. Clin. Periodontol..

[6] Avila E.D., Molon R.S., Barros-Filho L.A., Andrade M.F., Mollo F.A.J., Barros L.A. (2014). Correction of Malpositioned Implants through Periodontal Surgery and Prosthetic Rehabilitation Using Angled Abutment. Case Rep. Dent..

[7] Block M.S., Emery R.W., Cullum D.R., Sheikh A. (2017). Implant Placement Is More Accurate Using Dynamic Navigation. J. Oral Maxillofac. Surg..

[8] Chen W., Taezi K.A.A., Chu C.H., Shen Y., Wu J., Cai K., Chen P., Tang C. (2023). Accuracy of dental implant placement with a robotic system in partially edentulous patients: A prospective, single-arm clinical trial. Clin. Oral Implants Res..

[9] Maló P., Rangert B., Dvärsäter L. (2003). Function concept immediate “All-on-Four” with Brånemark System® Jaw Implants totally edentulous: A study clinical retrospective. Clin. Implant. Dent. Relat. Res..

[10] Park S.E., Silva D.J.D., Weber H., Ishikawa-Nagai S. (2007). Optical phenomenon of peri-implant soft tissue—Part I: Spectrophotometric assessment of natural tooth gingiva and peri-implant mucosa. Clin. Oral Implant. Res..

[11] Li K., Liu F., Liu P., Wei C., Li X. (2023). Clinical Effect and Aesthetic Evaluation of Minimally Invasive Implant Therapy. Emerg. Med. Int..

[12] Corsalini M., D’Agostino S., Favia G., Dolci M., Tempesta A., Di Venere D., Limongelli L., Capodiferro S. (2020). A Minimally Invasive Technique for Short Spiral Implant Insertion with Contextual Crestal Sinus Lifting in the Atrophic Maxilla: A Preliminary Report. Healthcare.

[13] Smitkarn P., Subbalekha K., Mattheos N., Pimkhaokham A. (2019). The accuracy of single-tooth implants placed using fully digital-guided surgery and freehand implant surgery. J. Clin. Periodontol..

[14] Kapos T., Evans C. (2014). CAD/CAM technology for implant abutments, crowns, and superstructures. Int. J. Oral. Maxillofac. Implants.

[15] Joda T., Wittneben J.G., Brägger U. (2014). Digital implant impressions with the “Individualized Scanbody Technique” for emergence profile support. Clin. Oral Implants Res..

[16] Morton D., Chen S.T., Martin W.C., Levine R.A., Buser D. (2014). Consensus statements and recommended clinical procedures regarding optimizing esthetic outcomes in implant dentistry. Int. J. Oral Maxillofac. Implants.

[17] Lops D., Parpaiola A., Paniz G., Sbricoli L., Magaz V.R., Venezze A.C., Bressan E., Stellini E. (2017). Interproximal Papilla Stability Around CAD/CAM and Stock Abutments in Anterior Regions: A 2-Year Prospective Multicenter Cohort Study. Int. J. Periodontics Restor. Dent..

[18] Marchack C.B. (1996). A custom titanium abutment for the anterior single-tooth implant. J. Prosthet. Dent..

[19] Benic G.I., Mir-Mari J., Hammerle C.H.F. (2014). Loading Protocols for Single-Implant Crowns: A Systematic Review and Meta-Analysis. Int. J. Oral Maxillofac. Implants.

[20] Neves F.D., Silveira-Júnior C.D., Coró V., Silva-Neto J.P., Simamoto-Júnior P.C., Prado C.J. (2013). Gingival conditioning in an implant-supported prosthesis: A clinical report. J. Oral Implantol..

[21] Mishra S.K., Chowdhary R., Chrcanovic B.R., Brånemark P.I. (2016). Osseoperception in Dental Implants: A Systematic Review. J. Prosthodont..

[22] Bornstein M.M., Balsiger R., Sendi P., von Arx T. (2011). Morphology of the nasopalatine canal and dental implant surgery: A radiographic analysis of 100 consecutive patients using limited cone-beam computed tomography. Clin. Oral Implants Res..

[23] Grunder U. (2011). Crestal ridge width changes when placing implants at the time of tooth extraction with and without soft tissue augmentation after a healing period of 6 months: Report of 24 consecutive cases. Int. J. Periodontics Restor. Dent..

[24] Arora H., Ivanovski S. (2018). Clinical and aesthetic outcomes of immediately placed single-tooth implants with immediate vs. delayed restoration in the anterior maxilla: A retrospective cohort study. Clin. Oral Implants Res..

[25] De Rouck T., Collys K., Cosyn J. (2008). Single-tooth replacement in the anterior maxilla by means of immediate implantation and provisionalization: A review. Int. J. Oral Maxillofac. Implants..

[26] Santosa R.E. (2007). Provisional restoration options in implant dentistry. Aust. Dent. J..

[27] Priest G. (2006). Esthetic potential of single-implant provisional restorations: Selection criteria of available alternatives. J. Esthet. Restor. Tooth..

[28] Berroeta E., Zabalegui I., Donovan T., Chee W. (2015). Dynamic Abutment: A method of redirecting screw access for implant-supported restorations: Technical details and a clinical report. J. Prosthet. Dent..

[29] Cavallaro J., Greenstein G. (2011). Angled implant abutments: A practical application of available knowledge. J. Am. Dent. Assoc..

[30] Ha C.Y., Lim Y.J., Kim M.J., Choi J.H. (2011). The influence of abutment angulation on screw loosening of implants in the anterior maxilla. Int. J. Oral Maxillofac. Implants.

[31] Imakita C., Shiota M., Yamaguchi Y., Kasugai S., Wakabayashi N. (2013). Failure analysis of an abutment fracture on single implant restoration. Implant Dent..

[32] Kitamura E., Stegaroiu R., Nomura S., Miyakawa O. (2004). Biomechanical aspects of marginal bone resorption around osseointegrated implants: Considerations based on a three-dimensional finite element analysis. Clin. Oral Implants Res..

[33] Rungtanakiat P., Thitaphanich N., Chengprapakorn W., Janda M., Arksornnukit M., Mattheos N. (2023). Association of prosthetic angles of the Implant Supracrestal Complex with peri-implant tissue mucositis. Clin. Exp. Dent. Res..

[34] Naert I., Duyck J., Vandamme K. (2012). Occlusal overload and bone/implant loss. Clin. Oral Implants Res..

[35] Di Fiore A., Montagner M., Sivolella S., Stellini E., Yilmaz B., Brunello G. (2022). Peri-Implant Bone Loss and Overload: A Systematic Review Focusing on Occlusal Analysis through Digital and Analogic Methods. J. Clin. Med..

[36] França D.G., Morais M.H., das Neves F.D., Barbosa G.A. (2015). Influence of CAD/CAM on the fit accuracy of implant-supported zirconia and cobalt-chromium fixed dental prostheses. J. Prosthet. Dent..

[37] Neves F.D., Prado C.J., Prudente M.S., Carneiro T.A., Zancopé K., Davi L.R. (2014). Micro-computed tomography evaluation of marginal fit of lithium disilicate crowns fabricated by using chairside CAD/CAM systems or the heat-pressing technique. J. Prosthet. Dent..

[38] das Neves F.D., de Almeida T.A.P.N., do Prado C.J., Prudente M.S., Zancopé K., Davi L.R. (2014). Micrometric precision of prosthetic dental crowns obtained by optical scanning and computer-aided design/computer-aided manufacturing system. J. Prosthet. Dent..

[39] das Neves F.D., do Prado C.J., Prudente M.S., Carneiro T.A., Zancope K., Davi L.R. (2015). Microcomputed tomography marginal fit evaluation of computer-aided design/computer-aided manufacturing crowns with different methods of virtual model acquisition. Gen. Dent..

[40] Carneiro T.A.P.N., Prado C.J., Prudente M.S., Zancope K., Davi L.R., Mendonca G., Cooper L.F., Soares C.J., Neves F.D. (2016). Micro CT analysis of in-office computer aided designed/computer aided manufactured dental restorations. Comput. Methods Biomech. Biomed. Eng. Imaging Vis..

[41] Fuster-Torres M.A., Albalat-Estela S., Alcañiz-Raya M., Peñarrocha-Diago M. (2009). CAD/CAM dental systems in implant dentistry: Update. Med. Oral Patol. Oral Cirugía Bucal.

[42] Patel N. (2010). Integrating three-dimensional digital technologies for comprehensive implant dentistry. J. Am. Dent. Assoc..

[43] Drago C., Saldarriaga R.L., Domagala D., Almasri R. (2010). Volumetric determination of the amount of misfit in CAD/CAM and cast implant frameworks: A multicenter laboratory study. J. Am. Int. J. Oral Maxillofac. Implants.

[44] Abduo J., Lyons K., Bennani V., Waddell N., Swain M. (2011). Fit of screw-retained fixed implant frameworks fabricated by different methods: A systematic review. Int. J. Prosthodont..

[45] Wesemann C., Muallah J., Mah J., Bumann A. (2017). Accuracy and efficiency of full-arch digitalization and 3D printing: A comparison between desktop model scanners, an intraoral scanner, a CBCT model scan, and stereolithographic 3D printing. Quintessence Int..

[46] Eftekhar A.R., Nasiri K.L., Mahshid M., Moshaverinia A. (2017). Comparison of dimensional accuracy of conventionally and digitally manufactured intracoronal restorations. J. Prosthet. Dent..

[47] Kim D.Y., Lee H.N., Kim J.H., Kim H.Y., Kim W.C. (2017). Evaluation of marginal and internal gaps in single and three-unit metal frameworks made by micro-stereolithography. J. Adv. Prosthodont..

[48] Revilla-León M., Gonzalez-Martín Ó., Pérez López J., Sánchez-Rubio J.L., Özcan M. (2018). Position Accuracy of Implant Analogs on 3D Printed Polymer versus Conventional Dental Stone Casts Measured Using a Coordinate Measuring Machine. J. Prosthodont..

[49] Tomita Y., Uechi J., Konno M., Sasamoto S., Iijima M., Mizoguchi I. (2018). Accuracy of digital models generated by conventional impression/plaster-model methods and intraoral scanning. Dent. Mater. J..

[50] Deeb G.R., Allen R.K., Hall V.P., Whitley D., Laskin D.M., Bencharit S. (2017). How Accurate Are Implant Surgical Guides Produced With Desktop Stereolithographic 3-Dimentional Printers?. J. Oral Maxillofac. Surg..

[51] Mangano F., Gandolfi A., Luongo G., Logozzo S. (2017). Intraoral scanners in dentistry: Review of the current literature. BMC Oral Health.

[52] Chen Z., Li J., Ceolin-Meneghetti P., Galli M., Mendonça G., Wang H.L. (2022). Does guided level (fully or partially) influence implant placement accuracy at post-extraction sockets and healed sites? An in vitro study. Clin. Oral Investig..

